# Effects of vascular endothelial growth factor-A and -C on wound healing in wild-type mice

**DOI:** 10.1371/journal.pone.0352811

**Published:** 2026-06-29

**Authors:** Yukari Nakajima, Kanae Mukai, Kimi Asano, Toshio Nakatani

**Affiliations:** 1 Faculty of Health Sciences, Institute of Medical, Pharmaceutical and Health Sciences, Kanazawa University, Kanazawa, Ishikawa, Japan; 2 School of Nursing, Kanazawa Medical University, Uchinada, Ishikawa, Japan; Longgang Otorhinolaryngology Hospital & Shenzhen Key Laboratory of Otorhinolaryngology, Shenzhen Institute of Otorhinolaryngology, CHINA

## Abstract

This study aimed to investigate whether periwound subcutaneous administration of vascular endothelial growth factor-A (VEGF-A), VEGF-C, or both promotes wound healing in wild-type (WT) mice. A total of 40 9-week-old male BALB/c mice were used in this study. Two circular full-thickness skin wounds were created. Mice were treated with VEGF-A on day 4 after wound creation (VEGF-A group), VEGF-C on day 7 after wound creation (VEGF-C group), or VEGF-A and VEGF-C (VEGF-A + C group). The control group received subcutaneous saline injections on days 4 and 7. Photographs were taken, the wound area was measured, and the covering materials were changed for 15 days. Lymphography was performed before wound harvest. Tissues were harvested 4, 7, 10, and 14 days after wound creation and stained for lymphatic and blood vessels. The wounds in each group healed almost identically. No significant differences were observed between the VEGF-A, VEGF-C, and VEGF-A + C groups and the control group throughout the observation period. Similarly, no significant difference in the number of lymphatic and blood vessels within the granulation tissue after wound creation was observed between the VEGF-A, VEGF-C, and VEGF-A + C groups and the control group. Collecting lymphatic vessels did not regenerate across the wound, whereas capillary lymphatic vessels regenerated within the wound. Endogenous repair responses in acute wounds in WT mice progress relatively rapidly, and angiogenesis and lymphangiogenesis are sufficiently induced without VEGF-A or VEGF-C. Therefore, additional exogenous VEGF-A or VEGF-C may provide little incremental benefit, consistent with a ceiling effect.

## Introduction

Cutaneous wound healing is a highly coordinated physiological process involving hemostasis, inflammation, proliferation (granulation tissue formation), and remodeling, and timely neovascularization is required to restore oxygen and nutrient delivery to the wound bed [1–3]. Vascular endothelial growth factor-A (VEGF-A) is a central proangiogenic cytokine, and VEGF receptor 2 is a key signaling receptor that mediates VEGF-driven angiogenesis in endothelial cells [4]. Lymphatic vessels contribute to fluid homeostasis and immune cell trafficking during wound repair. Lymphangiogenesis is a crucial component of cutaneous wound healing, and impaired lymphangiogenic responses are associated with delayed wound healing in chronic or metabolically compromised conditions [5]. VEGF-C plays a key role in lymphangiogenesis by activating VEGF receptor 3 in lymphatic endothelial cells, thereby promoting lymphatic vessel growth [6].

Given these biological roles, the therapeutic augmentation of VEGF signaling has most often been evaluated in conditions where endogenous repair is insufficient. In wounds in diabetic db/db mice, repeated administration of VEGF165, a splice variant of VEGF-A, has been reported to shorten the time to re-epithelialization and to enhance wound repair, accompanied by local upregulation of growth factors crucial for tissue regeneration [7]. Furthermore, studies using adenoviral vectors to overexpress VEGF-C have reported that VEGF-C markedly accelerates wound healing by promoting angiogenesis and lymphangiogenesis [8]. However, studies investigating the effects of VEGF-A and VEGF-C on wound healing in wild-type (WT) mice are limited. Our previous study [9] showed that neovascularization in granulation tissue was observed up to 5 days after wound creation and peaked on day 7 in WT mice. Additionally, we reported that new lymphatic vessels were first observed on day 7 after wound creation and peaked on day 11.

Therefore, this study aimed to determine whether the administration of VEGF-A, VEGF-C, or both promotes wound healing. We hypothesized that early increases in angiogenesis and subsequent lymphangiogenesis induced by VEGF-A or VEGF-C administration could further accelerate wound healing in WT mice.

## Methods

### Mice

A total of 40 9-week-old male BALB/c mice (Sankyo Lab Service Corporation, Inc., Toyama, Japan) were used in this study. Mice were allocated to the following experimental groups to avoid significant differences in body weight among groups prior to wound creation: control (n = 13), VEGF-A (n = 10), VEGF-C (n = 10), and VEGF-A + C (n = 7). Mice were individually caged in an air-conditioned room at 25°C ± 2°C, and the lights were kept on from 8:45 am to 8:45 pm. Water and pelleted food were provided ad libitum.

### Wounding

**Fig 1:**
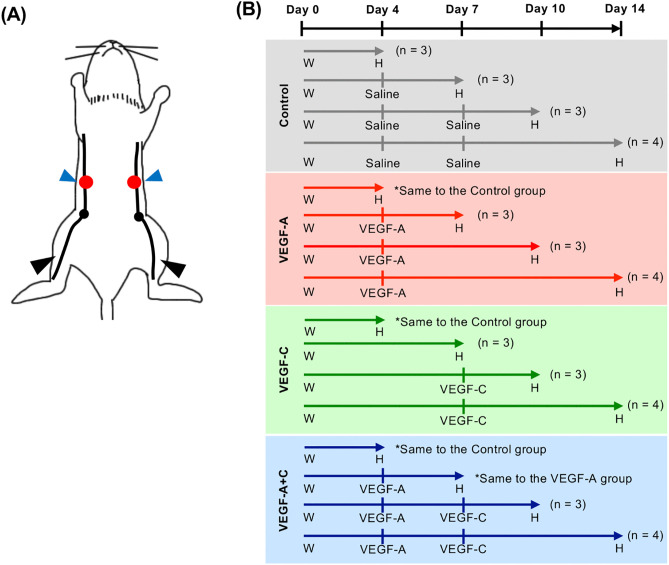
Wounding, treatment, and wound harvest time points for each group. **(A)** Schematic of mouse wounding. Wounds were made on the dorsal skin, 5 mm caudal to the subcostal level, including the collecting lymphatic and blood vessels. Red circles indicate wounds, black circles indicate lymph nodes, black lines indicate lymphatic vessels, black arrowheads indicate ICG injection sites, and blue arrowheads indicate VEGF-A, VEGF-C, and normal saline injection sites. **(B)** VEGF-A was administered on day 4, VEGF-C was administered on day 7, and normal saline was administered on days 4 and 7. All groups were under the same conditions on day 4, and the VEGF-A and VEGF-A + C groups were under identical conditions on day 7. n: number of mice; W: wounding; H: harvesting of wounds.

Mice were anesthetized with isoflurane inhalation anesthesia (1.5%–2%), and their dorsa were shaved. The following day, under inhalation anesthesia with isoflurane, a 27 G microsyringe was subcutaneously inserted on the medial side of the left and right lower limbs to administer 3 μL of indocyanine green (ICG) for lymphography as previously described [10]. Massage was performed around the injection site using a cotton swab to promote ICG absorption. Fluorescent images of the collecting lymphatic vessels from the inguinal to axillary lymph nodes were obtained using a near-infrared camera system (pde-neo; Hamamatsu Photonics, Shizuoka, Japan). The obtained images of collecting lymphatic vessels were marked with a black marker pen on the skin to detect them with the naked eye. Two 4-mm-diameter, full-thickness skin wounds were created on the dorsal skin, a few millimeters ventral to the frequently used dorsal wound site and 5 mm caudal to the subcostal level, using a biopsy punch (Kai Industries, Gifu, Japan) (Fig 1A). The wound site included the collecting lymphatic vessels and their accompanying blood vessels running through this region. The wound creation date was set to day 0.

Wounds were covered with a hydrocolloid dressing (Tegaderm^TM^, 3M Health Care, Maplewood, MN, USA), and the abdomen was wrapped with an ultra-hypoallergenic adhesive bandage (Skinergate^TM^, Nichiban Corporation, Tokyo, Japan). Macroscopic observation was performed daily in the morning under isoflurane inhalation anesthesia (1.5%), and the wound area was cleaned using cotton swabs and normal saline. The hydrocolloid dressing and bandage were changed daily until the day of wound retrieval.

### Administration of VEGF-A and VEGF-C

Shimamura et al. [8] reported that angiogenesis was observed in the granulation tissue on day 5 after wound creation and peaked on day 7, whereas lymphangiogenesis was first observed on day 7 after wound creation and peaked on day 11. We hypothesized that the administration of VEGF-A and VEGF-C on days 4 and 7, when angiogenesis and lymphangiogenesis began, respectively, could accelerate wound healing. The dosage of proliferating factors was determined based on previous studies [11,12] and a preliminary examination in which safety and wound closure rate were used as endpoints. After wound creation, the mice were divided into four groups: control, VEGF-A, VEGF-C, and VEGF-A + VEGF-C (VEGF-A + C).

Normal saline, VEGF-A, or VEGF-C was subcutaneously administered at a single point 5 mm dorsal to the wounds using a 27-G needle. In the control group, 100 μL of normal saline was administered on days 4 and 7 after wound creation. In the VEGF-A group, 250 ng/100 μL VEGF-A (Recombinant Human VEGF165, PeproTech, London, UK) diluted in saline was administered on day 4. In the VEGF-C group, 250 ng/100 μL VEGF-C (Recombinant Human VEGF-C Protein, R&D Systems, Minneapolis, MN, USA) diluted in saline was administered on day 7. In the VEGF-A + C group, 250 ng/100 μL VEGF-A was administered on day 4, followed by 250 ng/100 μL VEGF-C on day 7.

Wound samples were collected on days 4, 7, 10, and 14 following wound creation, according to the schedule presented in Fig 1B. In the control group, samples were collected on days 4, 7, 10, and 14, with collection on days 4 and 7 performed before saline administration. In the VEGF-A and VEGF-C groups, samples were collected on days 7, 10, and 14. In the VEGF-C group, sample collection on day 7 was performed before VEGF-C administration. In the VEGF-A + C group, sample collection was performed on days 10 and 14.

Fig 1B summarizes the treatment procedures and wound harvest time points, and the number of mice used at each wound harvest time points for each group. Data were obtained from the same group of mice as long as the experimental conditions were identical to minimize animal use. Particularly, on day 4, all groups were under the same conditions. Therefore, the results from the same mice were used for all group comparisons at this time point. On day 7, the VEGF-A and VEGF-A + C groups were under identical conditions. Therefore, the results from the same mice were used for these two groups.

### Lymphography

In addition to observation of the collecting lymphatic vessels before wounding, lymphography was performed before wound harvest under inhalation anesthesia with 1.5% isoflurane. 3 μL of ICG was administered to the left and right lower hindlimbs after observation on days 4, 7, 10, and 14, and lymphatic flow was assessed. Fluorescence images of the lymphatic flow were then obtained using an infrared observation camera system (pde-neo; Hamamatsu Photonics, Shizuoka, Japan) to confirm the lymphatic flow. Fluorescence images were saved as photographs.

### Macroscopic observation

Observation, photography, and measurement of the wound areas were performed in all groups in the morning for 15 days, from 0 to 14 days after wound creation. The wound area was measured by placing an overhead projector (OHP) sheet in direct contact with the wound and tracing the wound edges with an oil-based pen. The OHP sheet was scanned, and the wound area was measured using Scion Image Beta 4.02 (Scion Corporation, USA). The wound area on each day was expressed as a relative value, with the area on the day of wound creation being 1.

### Tissue processing

Mice were euthanized by overdose inhalation of isoflurane via an anesthesia system. Exposure was continued until respiratory arrest occurred, and death was confirmed by cessation of respiration and heartbeat. The area of the tissue sample, including the scar and surrounding normal tissue, was sectioned into rectangles of 25 mm × 25 mm. The harvested tissues were stapled onto a 30 mm × 30 mm sheet of OHP to prevent excessive section shrinkage and fixed in 4% paraformaldehyde/0.1 M phosphate buffer for 24 h at 4°C. They were washed with 0.01 M phosphate-buffered saline, embedded in paraffin, and sectioned into 5 μm thick serial sections using a microtome.

### Staining

Hematoxylin and eosin staining was performed to observe tissue morphology. For anti-von Willebrand factor (vWF) antibody immunostaining, deparaffinized sections were treated with Proteinase K for 10 min at room temperature. After washing with 0.03% Tween 20 in PBS, the sections were blocked with 5% bovine serum albumin. Subsequently, the sections were then incubated with the primary antibody against vWF (ab6994, Abcam, Cambridge, UK) at a 1:4000 dilution for 60 min at room temperature, followed by incubation with the secondary antibody (K4003, Dako, Glostrup, Denmark) for 30 min at room temperature. Immunoreactivity was visualized using 3,3′-diaminobenzidine. For anti-LYVE-1 antibody immunostaining, deparaffinized sections were blocked with 5% bovine serum albumin.After washing with 0.03% Tween 20 in PBS, the sections were incubated with the primary antibody against LYVE-1 (ab14917, Abcam, Cambridge, UK) at a 1:2000 dilution overnight at 4°C, followed by incubation with the secondary antibody (P0399, Dako, Glostrup, Denmark) at a 1:600 dilution for 30 min at room temperature. Immunoreactivity was visualized using 3,3′-diaminobenzidine.

### Number of lymphatic and blood vessels

Stained images were obtained using a digital microscope camera (DP27-CU; Evident, Tokyo, Japan) and transferred to a computer. Blood and lymphatic vessels were quantified at an original magnification of 200 × . The number of blood or lymphatic vessels was counted in 1–3 wound areas, divided by the measured area, and expressed as the number per mm^²^.

### Ethical statement

All procedures involving animals were reviewed and approved by the Committee on Animal Experimentation of Kanazawa University (AP–194078) and conducted in accordance with the approved guidelines.

### Statistical analyses

Data were presented as mean ± standard error of the mean (SEM). Data analysis was performed using GraphPad Prism 10 (GraphPad Software, Boston, MA, USA). To avoid pseudoreplication, the mouse was treated as the statistical unit for analysis. Therefore, the values from the two wounds in each mouse were averaged and used as one mouse-level value for statistical analysis. The means of multiple groups were compared using one-way analysis of variance (ANOVA) or the Kruskal–Wallis test. Post hoc pairwise comparisons were performed using Tukey’s multiple comparison test or Dunn’s multiple comparison test. A *p*-value <0.05 indicated statistical significance.

## Results

### Macroscopic observation

**Fig 2:**
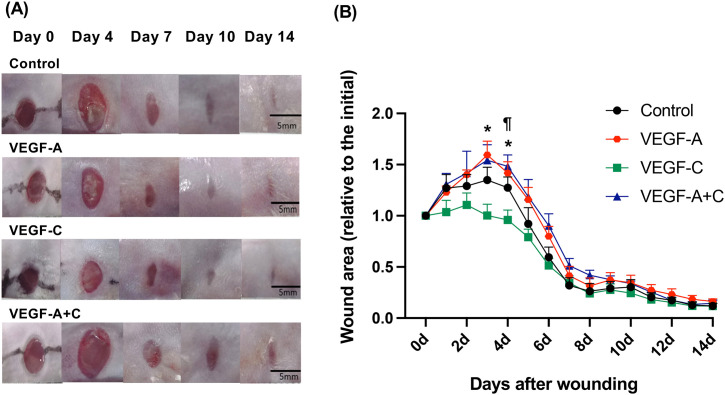
Representative images of the wound and the ratio of the wound area. **(A)** Images of wounds in each group. Scale bar, 5 mm. **(B)** Ratio of the wound area to the initial area as line graphs for each day. Values are presented as means ± SEM, *n* = 4 mice in all groups. ANOVA with Tukey’s multiple comparison test, ^*^p < 0.05: VEGF-A vs. VEGF-C; ^¶^*p* < 0.05: VEGF-C vs. VEGF-A + C.

Granulation tissue appeared approximately 4 days after wound creation, and the wound area gradually decreased. It was covered by the epidermis on day 10, and a scar was formed 14 days after wound creation (Fig 2A). The granulation tissue appeared reddened during the observation period. No marked edema was observed at the wound site or its surrounding area. The wound area in the VEGF-C group peaked on day 2, and in the control, VEGF-A, and VEGF-A + C groups peaked on day 3 after wound creation. On day 3, the VEGF-C group exhibited a significantly smaller wound area than the VEGF-A groups (*p* = 0.0337). On day 4, the VEGF-C group exhibited a significantly smaller wound area than the VEGF-A + C and VEGF-A groups (*p* = 0.0205 and *p* = 0.0433, respectively) (Fig 2B). No significant differences were observed between the control and the VEGF-A groups, between the control and the VEGF-C groups, or between the control and the VEGF-A + C groups throughout the observation period.

### Lymphography

**Fig 3:**
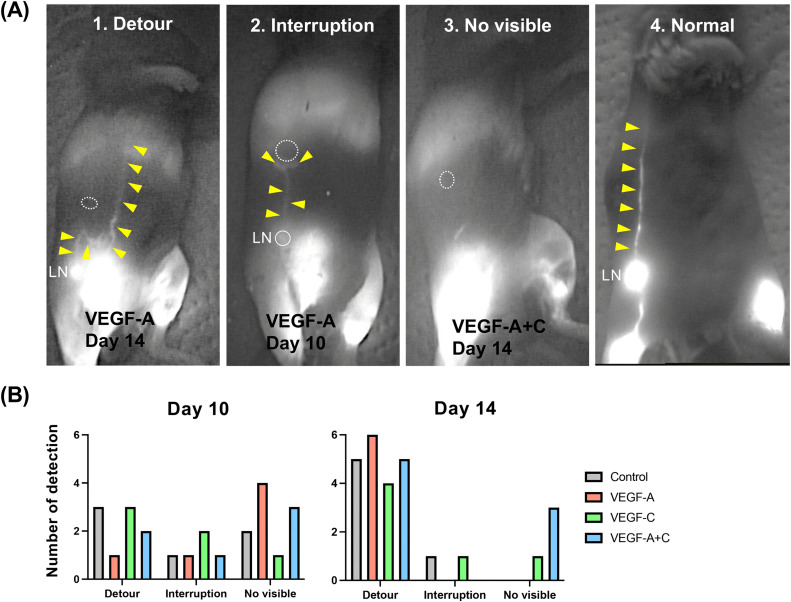
Lymphography. **(A)** Representative images of lymphography: detour in the VEGF-A group on day 14, interruption in the VEGF-A group on day 10, no visible in the VEGF-A + C group on day 14, and normal before wound creation. LN: lymph node; white circle: LN location; yellow arrow: lymph flow; dotted circle: wound location. **(B)** Number of lymphography detections on days 10 and 14. *n* = 3 mice in the control, VEGF-A, VEGF-C, and VEGF-A + C groups.

**Fig 4:**
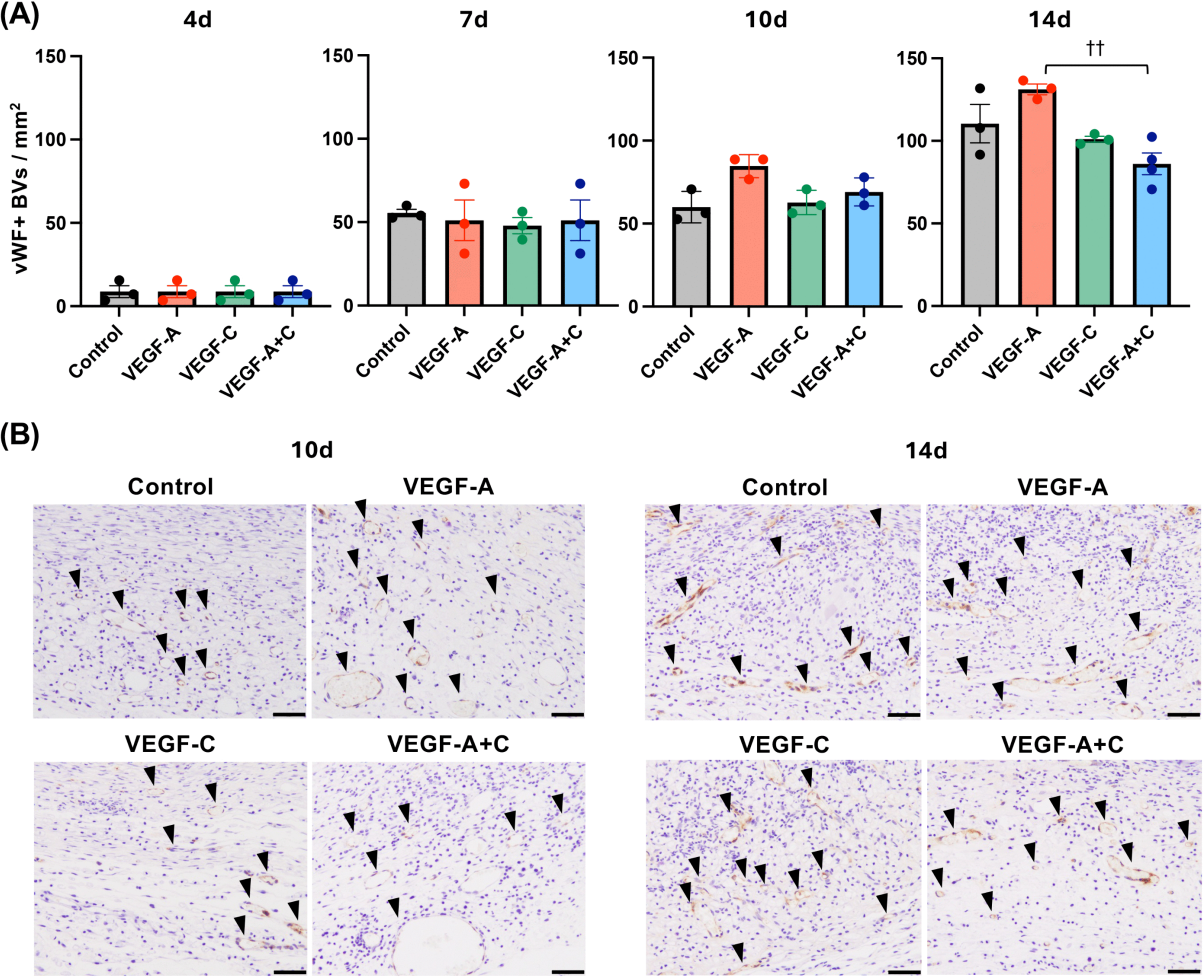
Blood vessels. **(A)** Number of blood vessels/mm^2^ on days 4, 7, 10, and 14. Values are presented as means ± SEM, *n* = 3–4 mice in each group. ANOVA with Tukey’s multiple comparisons test: days 4, 7, and 14, ^††^*p* < 0.01: VEGF-A vs. VEGF-A + C. Kruskal–Wallis test: day 10. vWF: von Willebrand factor, BV: blood vessel. **(B)** Anti-vWF antibody immunohistochemistry staining on days 10 and 14. Arrowheads indicate vWF^+^ blood vessels. Scale bar, 50 µm.

Collecting lymphatic vessels were observed from the lower limbs through the inguinal lymph nodes to the axillary region in all mice before wound creation (Fig 3A-4). After wound creation, including the collecting lymphatic vessels, lymphatic routing was evaluated via ICG lymphography, identifying three distinct imaging patterns. Pattern 1 was a detour (bypass) pattern (Fig 3A-1): after passing through the inguinal lymph node, the ICG signal detoured either ventrally or dorsally before reaching the wound site, draining into adjacent collecting lymphatic vessels. Pattern 2 was an interruption pattern (Fig 3A-2): after passing through the inguinal lymph node, the ICG signal ascended directly to reach the area immediately proximal to the wound. No lymphatic flow was observed cranially to the wound, with ICG pooling/accumulation observed around the wound margin. Pattern 3 was no visible pattern (Fig 3A-3). ICG was administered to the hind limb, and lymphography was performed using the same procedure in all mice. However, no lymphatic vessels were visualized in some mice, even on postoperative day 14. Additionally, no ICG signal traversing the wound site was observed. A detour was formed in most mice by day 14 (Fig 3B). An interruption pattern was observed in one mouse in the control and VEGF-C groups. No visible lymphatic vessel pattern was observed in one mouse in the VEGF-C group and in three mice in the VEGF-A + C group. ICG signals were not clearly visualized in most mice on days 4 and 7.

### Blood vessels

Angiogenesis was observed in the granulation tissue on day 4 (Fig 4). Angiogenesis remained active through day 14 and peaked on that day. On day 14, the VEGF-A group exhibited a significantly higher number of blood vessels than the VEGF-A + C group (*p* = 0.0051).

### Lymphatic vessels

**Fig 5:**
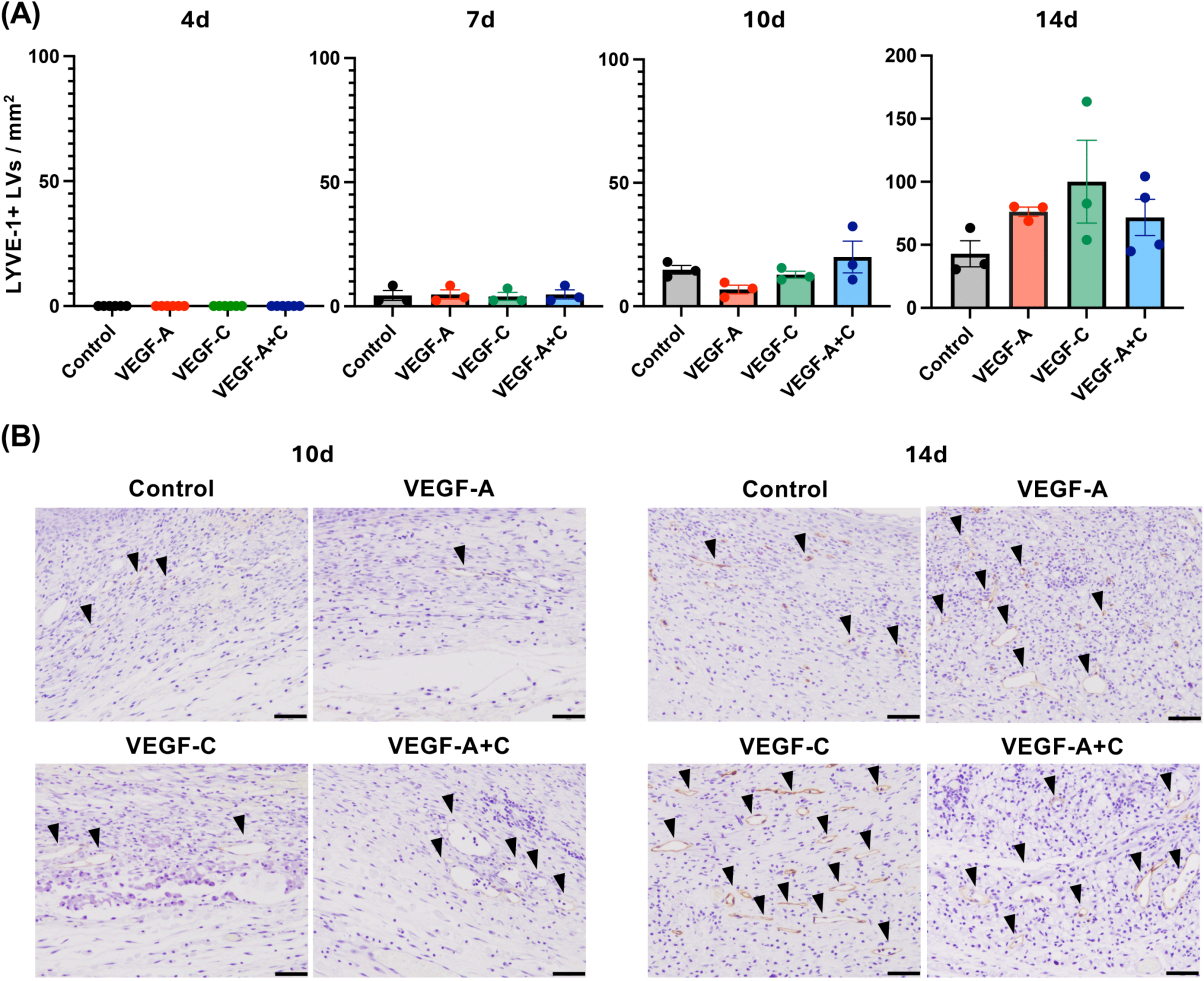
Lymphatic vessels. **(A)** Number of lymphatic vessels/mm^2^ on days 4, 7, 10, and 14. Values are presented as means ± SEM, *n* = 3–4 mice in each group. ANOVA: days 4, 10, and 14; Kruskal–Wallis test: day 7. LVs: lymphatic vessels. **(B)** Anti-LYVE-1 antibody immunohistochemistry staining on days 10 and 14. Arrowheads indicate LYVE-1^+^ lymphatic vessels. Scale bar, 50 µm.

On day 4, no lymphangiogenesis was observed (Fig 5). Lymphangiogenesis was first observed in the granulation tissue on day 7. The number of lymphatic vessels on day 10 increased compared with that on day 7. The number of lymphatic vessels increased dramatically between days 10 and 14, with the number rising by approximately threefold in the control group, tenfold in the VEGF-A group, eightfold in the VEGF-C group, and threefold in the VEGF-A + C group. Nosignificant differences were observed during the observation period.

## Discussion

A cutaneous wound model in WT mice was used in this study. VEGF-A was administered to promote angiogenesis by subcutaneous periwound injection on day 4 after wounding, and VEGF-C was administered to promote lymphangiogenesis by subcutaneous periwound injection on day 7. We hypothesized that increasing blood and lymphatic vessels in the granulation tissue would accelerate the healing process and promote wound closure. However, no significant differences in wound area or angiogenesis/lymphangiogenesis within the granulation tissue were observed between the VEGF-A, VEGF-C, and VEGF-A + C groups and the control group. These findings indicate that endogenous repair responses progress relatively rapidly in acute wounds in WT mice, and angiogenesis and lymphangiogenesis are sufficiently induced. Therefore, additional exogenous VEGF-A and VEGF-C may provide little incremental benefit, consistent with a ceiling effect.

The effectiveness of VEGF-based interventions has been demonstrated under conditions associated with impaired healing. In db/db mouse skin wounds, repeated administration of VEGF-A, every other day (20 μg per dose, 5 doses) has been reported to shorten the time to reepithelialization and enhance wound repair, with local upregulation of growth factors important for tissue regeneration [7]. Similarly, in db/db mouse skin wounds, VEGF-C has been reported to markedly accelerate wound healing by promoting angiogenesis and lymphangiogenesis [8]. Compared with these delayed healing models, acute wounds in WT mice may exhibit robust healing and high levels of angiogenic and lymphangiogenic responses, making further enhancement of wound repair difficult.

Additionally, a single-dose regimen for VEGF-A and VEGF-C was adopted. Previous studies were designed to maintain sustained local exposure. Repeated dosing was used for VEGF-A protein, and adenoviral delivery was used for VEGF-C to achieve prolonged local expression [8]. Accordingly, a single injection may result in a shorter duration of effective local exposure compared with these sustained-delivery approaches. In this study, repeated injections were not performed to minimize the invasiveness associated with subcutaneous administration and reduce puncture-related local inflammation, thereby enabling the evaluation of VEGF effects with minimal procedural confounding. Furthermore, excessive or prolonged VEGF stimulation can induce increased vascular permeability and edema. Continuous VEGF expression in conditional transgenic models drives persistent angiogenesis, formation of aberrant vessels, and increased edema [13]. High VEGF-C concentrations have been reported to enlarge vessel diameter and increase vascular leakage [14]. No obvious adverse events attributable to VEGF administration were observed under our protocol with fewer injections than in sustained-delivery studies. Notably, no significant inflammation or edema was observed during the observation period.

Our previous study [9] showed that the peak of angiogenesis in acute wounds in WT mice occurred on day 7, whereas the peak of lymphangiogenesis occurred on day 11. However, the peaks of angiogenesis and lymphangiogenesis in this study occurred on day 14 in all groups, contradicting our previous findings. This discrepancy may be due to the periwound injection procedure. Although the number of injections was minimized, two in the control and VEGF-A + C groups and one in the VEGF-A and VEGF-C groups, periwound puncture may have influenced the wound microenvironment and affected the kinetics of angiogenic and lymphangiogenic responses. However, it did not delay wound closure. Less invasive delivery strategies are preferable from a methodological perspective. The development of less invasive approaches is essential for future studies assessing the effects of VEGF on wound repair.

The limited efficacy of VEGF-A and VEGF-C administration in this study does not indicate that VEGF signaling is not involved in acute wound healing in WT mice. Considering that the rapid healing process in WT mice might have obscured a potential wound-healing-promoting effect is important.

In this study, wound creation involved excising the collecting lymphatic vessels and the accompanying blood vessels within the subcutaneous tissue. Angiogenesis was observed within the granulation tissue beginning on day 4. Granulation tissue appeared red, no finding suggestive of reduced perfusion was observed, and wound healing was not delayed. Lymphangiogenesis began later than angiogenesis, starting on day 7. Histological analysis did not identify collecting lymphatic vessels within the wounds, and lymphography did not detect collecting lymphatic vessels traversing the wound area. Lymph flow appeared to form detours or be interrupted proximal to the wound. Ligation of collecting lymphatic vessels at two sites in the abdomen has been reported to abolish lymph flow across the ligated segment and result in detour formation without collecting vessel regeneration [15]. These findings indicate that collecting lymphatic vessels does not regenerate even after mechanical injury. Conversely, capillary lymphatic vessels regenerated within the wound area, and no significant edema was observed in the wound or surrounding tissue. These findings indicate that lymphatic drainage in and around the wound is mainly dependent on capillary lymphatic vessels. The lymph may be drained without excessive fluid accumulation, allowing wound healing to proceed without delay.

This study has several limitations. First, a reference drug or positive control that promotes wound healing, angiogenesis, or lymphangiogenesis was excluded. Therefore, the effects of VEGF-A and/or VEGF-C could only be compared with those of the vehicle control. Second, local VEGF levels were not quantified after injection. Therefore, when and to what extent local VEGF blood concentrations increased remains unclear. This lack of exposure confirmation limits the interpretation of the negative findings, as we could not determine whether VEGF-A and/or VEGF-C reached and maintained sufficient local concentrations to exert biological effects in the wound microenvironment. Thus, the negative findings may reflect either a ceiling effect in acute wounds in WT mice or insufficient local exposure following single-dose administration. Third, because the primary endpoints focused on macroscopic wound area and the number of vessels within granulation tissue, the potential contribution of wound contraction to wound closure could not be distinguished from re-epithelialization. As contraction dominates closure of murine excisional wounds, it may have obscured subtle effects of VEGF-A and/or VEGF-C on angiogenesis or wound healing. In addition, quantitative histological assessment of re-epithelialization, such as epithelial gap length or epithelial coverage percentage, was not performed, and differences in vascular or lymphatic function, such as permeability, lymphatic drainage capacity, and vascular maturity, could not be verified. Fourth, all experiments were conducted using an acute wound model in WT mice, which may limit the generalizability of the findings to conditions with delayed healing, such as diabetes and aging. Fifth, injection into the wound periphery might have influenced the wound microenvironment and the dynamics of the angiogenesis/lymphangiogenesis response. Additional approaches are required to definitively elucidate the effects of VEGF-A and VEGF-C on wound healing in WT mice.

In conclusion, periwound subcutaneous administration of VEGF-A, VEGF-C, or both in WT mice did not significantly decrease wound area or increase angiogenesis/lymphangiogenesis compared with the control group. These findings may reflect a ceiling effect in acute wounds in WT mice, in which endogenous repair and vascular/lymphatic responses are robust without additional exogenous VEGF-A and VEGF-C. Compared with sustained-delivery regimens, a single-dose administration may have provided insufficient local exposure while avoiding overt adverse events, such as abnormal structure and significant edema. Collecting lymphatic vessels did not regenerate across the wound, whereas capillary lymphatic vessels regenerated within the wound. Acute wound healing proceeded without delay, indicating that capillary lymphatics may be sufficient to maintain lymphatic drainage in mice even when collecting vessels are disrupted.

## References

[1] Martin P. Wound healing—aiming for perfect skin regeneration. Science. 1997;276:75–81. doi: 10.1126/science.276.5309.75 9082989

[2] Singer AJ, Clark RA. Cutaneous wound healing. N Engl J Med. 1999;341(10):738–46. doi: 10.1056/NEJM199909023411006 10471461

[3] Saoudi M, Badraoui R, Chira A, Saeed M, Bouali N, Elkahoui S, et al. The Role of Allium subhirsutum L. in the Attenuation of Dermal Wounds by Modulating Oxidative Stress and Inflammation in Wistar Albino Rats. Molecules. 2021;26(16):4875. doi: 10.3390/molecules26164875 34443463 PMC8398921

[4] Shah FH, Nam YS, Bang JY, Hwang IS, Kim DH, Ki M, et al. Targeting vascular endothelial growth receptor-2 (VEGFR-2): structural biology, functional insights, and therapeutic resistance. Arch Pharm Res. 2025;48(5):404–25. doi: 10.1007/s12272-025-01545-1 40341988 PMC12106596

[5] Renò F, Sabbatini M. Breaking a Vicious Circle: Lymphangiogenesis as a New Therapeutic Target in Wound Healing. Biomedicines. 2023;11(3):656. doi: 10.3390/biomedicines11030656 36979635 PMC10045303

[6] Korhonen EA, Murtomäki A, Jha SK, Anisimov A, Pink A, Zhang Y, et al. Lymphangiogenesis requires Ang2/Tie/PI3K signaling for VEGFR3 cell-surface expression. J Clin Invest. 2022;132(15):e155478. doi: 10.1172/JCI155478 35763346 PMC9337826

[7] Galiano RD, Tepper OM, Pelo CR, Bhatt KA, Callaghan M, Bastidas N, et al. Topical vascular endothelial growth factor accelerates diabetic wound healing through increased angiogenesis and by mobilizing and recruiting bone marrow-derived cells. Am J Pathol. 2004;164(6):1935–47. doi: 10.1016/S0002-9440(10)63754-6 15161630 PMC1615774

[8] Saaristo A, Tammela T, Farkkilā A, Kärkkäinen M, Suominen E, Yla-Herttuala S, et al. Vascular endothelial growth factor-C accelerates diabetic wound healing. Am J Pathol. 2006;169(3):1080–7. doi: 10.2353/ajpath.2006.051251 16936280 PMC1698814

[9] Shimamura K, Nakatani T, Ueda A, Sugama J, Okuwa M. Relationship between lymphangiogenesis and exudates during the wound-healing process of mouse skin full-thickness wound. Wound Repair Regen. 2009;17(4):598–605. doi: 10.1111/j.1524-475X.2009.00512.x 19614925

[10] Komatsu E, Nakajima Y, Mukai K, Urai T, Asano K, Okuwa M, et al. Lymph Drainage During Wound Healing in a Hindlimb Lymphedema Mouse Model. Lymphat Res Biol. 2017;15(1):32–8. doi: 10.1089/lrb.2016.0026 28151088

[11] Greenberg JI, Shields DJ, Barillas SG, Acevedo LM, Murphy E, Huang J, et al. A role for VEGF as a negative regulator of pericyte function and vessel maturation. Nature. 2008;456(7223):809–13. doi: 10.1038/nature07424 18997771 PMC2605188

[12] Hall MA, Robinson H, Chan W, Sevick-Muraca EM. Detection of lymphangiogenesis by near-infrared fluorescence imaging and responses to VEGF-C during healing in a mouse full-dermis thickness wound model. Wound Repair Regen. 2013;21(4):604–15. doi: 10.1111/wrr.12063 23758174

[13] Dor Y, Djonov V, Abramovitch R, Itin A, Fishman GI, Carmeliet P, et al. Conditional switching of VEGF provides new insights into adult neovascularization and pro-angiogenic therapy. EMBO J. 2002;21(8):1939–47. doi: 10.1093/emboj/21.8.1939 11953313 PMC125962

[14] Saaristo A, Veikkola T, Enholm B, Hytönen M, Arola J, Pajusola K, et al. Adenoviral VEGF-C overexpression induces blood vessel enlargement, tortuosity, and leakiness but no sprouting angiogenesis in the skin or mucous membranes. FASEB J. 2002;16(9):1041–9. doi: 10.1096/fj.01-1042com 12087065

[15] Asano K, Nakajima Y, Mukai K, Urai T, Okuwa M, Sugama J, et al. Pre-collecting lymphatic vessels form detours following obstruction of lymphatic flow and function as collecting lymphatic vessels. PLoS One. 2020;15(1):e0227814. doi: 10.1371/journal.pone.0227814 31940420 PMC6961945

